# Private Asylums

**Published:** 1852-01-01

**Authors:** 


### PRIVATE ASYLUMS.

Several correspondents have written to us, complaining of the unprofessional conduct of a few proprietors of private asylums. This is a subject into which we cannot now fully enter. We have no hesitation, however, in expressing our concurrence in the view taken by nearly all who have addressed us on the subject, and of protesting against the quackish mode which some adopt to puff themselves and their establishments into temporary notoriety. The system pursued by these men is, we admit, calculated to injure materially the character of *all* private establishments, to limit their sphere of usefulness, and to degrade the proprietors in public and professional estimation. All respectable men should set their faces against the disgusting practice referred to. If a man imagines that by investing 300*l.* or 400*l.* a-year in puffing advertisements, he will be able to escape from his legitimate insignificance, and fill his asylum with patients, he will find, to his cost, that he has much overrated both the credulity of the public and profession. Unless proceedings like these are discountenanced, men of character, experience, and delicacy of feeling, will retire altogether from the management of these institutions, and they will be left solely to the conduct of any ignorant monied adventurer who may consider this a good mode of investing his capital. The profession, as a body, should refuse its support to men who by their proceedings thus degrade an honourable professional occupation. A correspondent has referred to the proceedings of one proprietor of a private asylum, who is in the habit of visiting occasionally provincial towns, calling upon the resident medical men, and introducing himself and puffing his establishment. In some instances, where this person is refused admission into the hall, he satisfies himself with impudently pushing under the front door a large card, upon which is engraved a sketch of his "splendid establishment," with a quantity of letter-press descriptive of the wonderful capabilities of himself and his house. This man has, in his proceedings, gone somewhat in advance of "Professor Holloway" and "Messrs. Morison and Moat;" for these pill-mongers satisfy themselves with advertising their nostrums, whilst the party to whom we refer travels about the country like a hawker in search of stray lunatics. We have heard of a London physician of some standing repudiating, in indignant language, the assumption that he was "specially engaged in the treatment of the insane;" and we heard a physician, also of position, say, that he should consider it less degrading to keep a public-house than an asylum.

Why should this feeling exist? Is it not in the main owing to the disreputable proceedings of a *few* illiterate pseudo-medical men who have embarked in this speciality, with no other object than that of self-aggrandizement? If medical men, of whose sagacity, learning, and even existence, the profession and public are, alas! in a state of lamentable ignorance, think it necessary to advertise themselves and their asylums, they should do so decently. The occasional announcement of the name and locality of the house ought to be deemed sufficient; but when they attempt to throw Mr. Robins in the shade by offensive puffs of themselves and exaggerated descriptions of their establishments, and do this continually, it cannot prove otherwise than derogatory and degrading to the profession. To the public we say, beware of the men and asylums thus constantly obtruded upon your notice. The profession will, we have no doubt, exercise a just discrimination in the matter.

